# Mechanism of action and therapeutic effects of oxidative stress and stem cell-based materials in skin aging: Current evidence and future perspectives

**DOI:** 10.3389/fbioe.2022.1082403

**Published:** 2023-01-09

**Authors:** Huan Qian, Yihan Shan, Ruicheng Gong, Danfeng Lin, Mengwen Zhang, Chen Wang, Lu Wang

**Affiliations:** ^1^ Department of Plastic Surgery, The Second Affiliated Hospital, School of Medicine, Zhejiang University, Hangzhou, China; ^2^ Wenzhou Medical University, Wenzhou, China; ^3^ Starbody plastic surgery Clinic, Hangzhou, China; ^4^ Department of Breast Surgery, The First Affiliated Hospital of Wenzhou Medical University, Wenzhou, China

**Keywords:** skin, aging, stem cell, oxidative stress, materials

## Abstract

Aging is associated with multiple degenerative diseases, including atherosclerosis, osteoporosis, and Alzheimer’s disease. As the most intuitive manifestation of aging, skin aging has received the most significant attention. Skin aging results from various intrinsic and extrinsic factors. Aged skin is characterized by wrinkles, laxity, elastosis, telangiectasia, and aberrant pigmentation. The underlying mechanism is complex and may involve cellular senescence, DNA damage, oxidative stress (OS), inflammation, and genetic mutations, among other factors. Among them, OS plays an important role in skin aging, and multiple antioxidants (e.g., vitamin C, glutathione, and melatonin) are considered to promote skin rejuvenation. In addition, stem cells that exhibit self-replication, multi-directional differentiation, and a strong paracrine function can exert anti-aging effects by inhibiting OS. With the further development of stem cell technology, treatments related to OS mitigation and involving stem cell use may have a promising future in anti-skin aging therapy.

## 1 Introduction

Aging is currently defined as a progressive disorder of tissue and organ functions over time, which eventually leads to numerous chronic pathologies (Calcinotto et al., 2019). Skin aging can be categorized into intrinsic aging and extrinsic aging (primarily photoaging) (Wu et al., 2019). Intrinsic aging occurs unavoidably as a result of chronological aging, whereas extrinsic aging occurs upon exposure to environmental factors, such as pollutants and ultraviolet light (Kohl et al., 2011). Both intrinsic and extrinsic aging damage the skin structure and cause dysfunction, which leads to wrinkling, hair loss, reduction of elasticity, impairment of the epidermal barrier maintenance, and multiple physical and psychological issues. In addition, aged skin is more susceptible to injury and infection, poor wound healing, and primary skin cancer (Giangreco et al., 2008). Oxidative stress (OS), a phenomenon characterized by an imbalance between reactive oxygen species (ROS) and antioxidants, has long been considered one of the major driving forces of accelerated skin aging and diseases (Velarde et al., 2012). Engendered by various sources (such as mitochondrial respiration, UV light exposure, and environmental pollution), the excessive accumulation of ROS can induce inflammation, cellular senescence, and aging in the skin (Sies et al., 2017). Skin rejuvenation, as an attempt to reverse the visible signs of aging, involves protection from OS (Yang et al., 2020).

Overall, the skin contains approximately 20 different types of cells (e.g., keratinocytes, melanocytes, Langerhans cells, fibroblasts, and others) which are constantly replenished by various stem cells (Blanpain and Fuchs, 2006). Skin stem cells can be further categorized as epidermal stem cells, hair follicle stem cells, dermal stem cells, and sebaceous gland stem cells (Zouboulis et al., 2008). Despite the tiny proportion they constitute (1%–10% in the basal layer of the epidermis and 0.3% of dermal foreskin fibroblasts), stem cells are highly valued for their self-renewal potential (Niemann and Watt, 2002). Findings from studies have shown the depletion of stem cells during aging (Youn et al., 2004; Aleemardani et al., 2021).

Well known for their beneficial effects on wound healing and skin rejuvenation, skin stem cell-based therapy is being investigated extensively (Dahl, 2012). For example, adipose-derived stem cells (ADSCs) are frequently used in regenerative medicine owing to their extensive paracrine activity, role in angiogenesis and immune modulation, and anti-oxidative potential (Kusuma et al., 2017). In this paper, we review the relationship between OS exposure-related skin aging and the role of stem cells, with an aim to provide novel strategies.

## 2 Mechanisms underlying skin aging

### 2.1 Skin aging

As the largest and one of the most complex organs, the skin primarily comprises three parts: epidermis, dermis, and subcutaneous tissue (Campbell et al., 2004). The epidermal layer is further divided into cornified, granular, spinous, and basal layers and contains keratinocytes (accounting for approximately 95% of all cells), melanocytes, and Langerhans cells (Eckhart and Zeeuwen, 2018). Connected by the dermal-epidermal junction, the dermis is mostly composed of fibroblasts and their secretory protein, the extracellular matrix (ECM). According to a single-cell analysis led by Zou *et al.*, the inactivation of HES1 in fibroblasts and KLF6 in keratinocytes can cause cellular senescence (Zou et al., 2021). The reduction of physical interaction between fibroblasts and keratinocytes is suggested to be associated with the impairment of epidermal stem cell maintenance in aged skin (Gruber et al., 2020). Adipocytes are localized to the subcutaneous tissue (Author Anonyms, 2017). Both intrinsic and extrinsic aging leads to changes in all three layers (especially the former two).

Intrinsic aging is a normal physiological process regulated by various genetic factors. It is characterized by thinning, dryness, laxity, fine wrinkles, decreased elasticity, increased brittleness, and susceptibility to several skin disorders such as fibroma mole and seborrheic keratosis (Lavker et al., 1986; Gilchrest, 1989; Gu et al., 2020). Histologically, keratinocytes proliferate at a slow pace and become less active, and the number of epidermal stem cells reduces (Gu et al., 2020). Meanwhile, cytoheterogeneity is more frequent in the basal region with lost polarity (Brégégère et al., 2003). The interface between the epidermis and dermis flattens, making the skin less tolerant to shearing forces (Lavker et al., 1987). Underneath, the decreased activity of fibroblasts is observed with the reduced production of collagen and elastin.

Long-term exposure to noxious pollutants (such as ozone, particulate matter, and cigarette smoke), ultraviolet radiation (UVR), and an unhealthy lifestyle accelerate skin aging (Burke, 2018; Pecorelli et al., 2019). Among them, UVR is the most common cause of photoaging and the primary reason for extrinsic aging (Cavinato and Jansen-Dürr, 2017). Unlike intrinsic aging, photoaging is characterized by pachulosis, thickness, laxity, deep and thick wrinkle formation, hyperpigmentation, telangiectasis, and a higher risk of malignancy (Gu et al., 2020). Histologically, one of the most prominent characteristics is the accumulation of amorphous elastic fibers and disordered collagen formation (Varani et al., 2004). Other typical changes include the flattening of endothelial cells, dilation of remaining skin vessels, and the redistribution of skin adipose (Hughes et al., 2004).

### 2.2 Involvement of OS in skin aging

**FIGURE 1:**
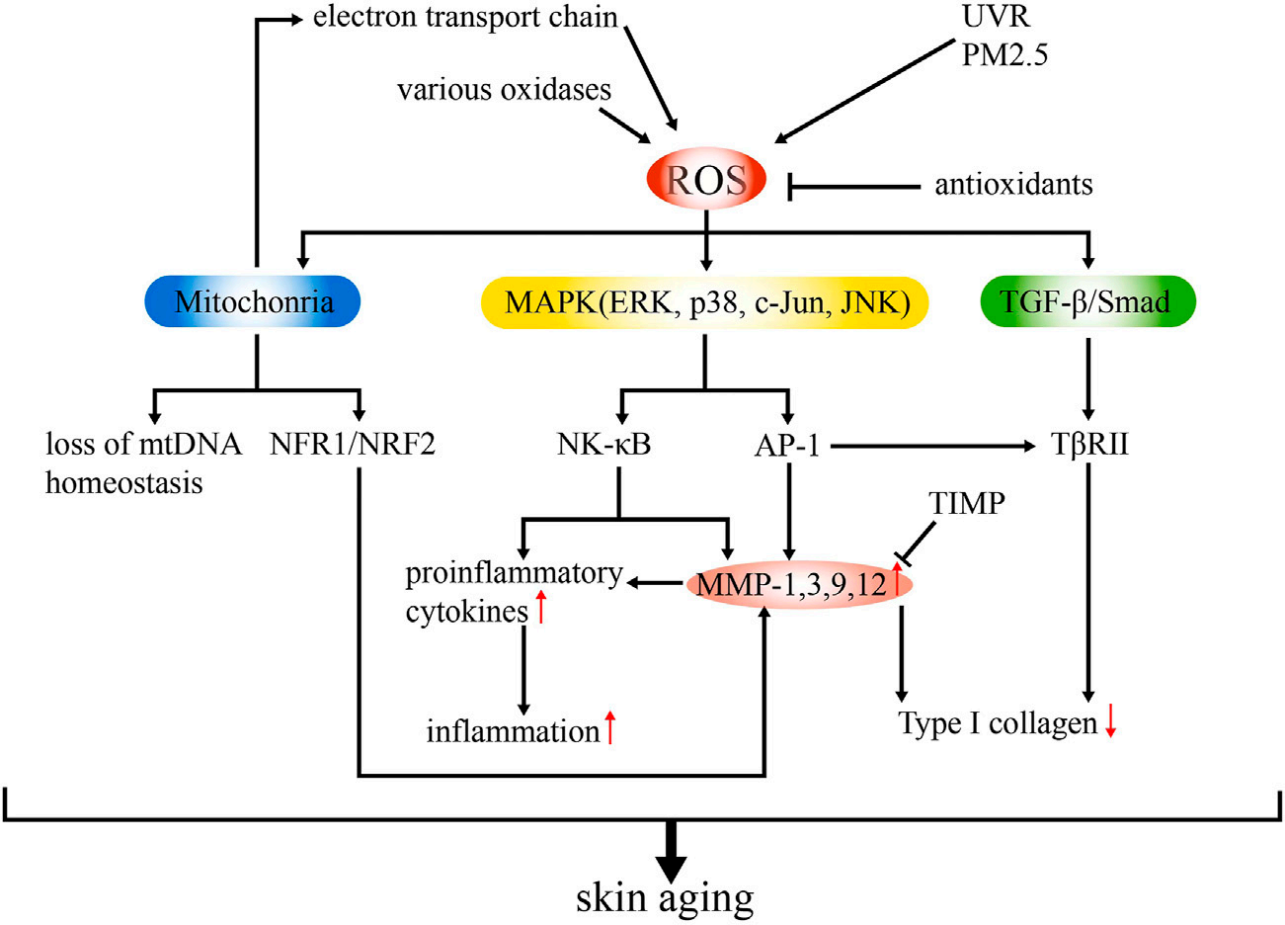
Schematic model of the mechanisms of ROS-related skin aging. ROS are generated from various intrinsic (e g., electron transport chain, various oxidases) and extrinsic sources (e.g., UVR, PM2.5). Overproduction of ROS can lead to upregulated MMP expression and inflammation and suppressed collagen synthesis *via* mitochondrial dysfunction and activation of MAPK and TGF-β signaling pathways. Antioxidants and TIMP can ameliorate ROS production, thus delaying skin aging.

The mechanisms underlying skin aging involve cellular, molecular, and genetic changes. Among them, OS is usually considered as the core influencing factor, exerting a key role in both intrinsic aging and photoaging (during which the effect is strengthened) through multiple signaling pathways and subsequent structural remodeling (Figure 1) (Bocheva et al., 2019; Prasanth et al., 2019).

OS results from redox imbalances, which involve ROS accumulation and antioxidant effect suppression (Birch-Machin and Bowman, 2016). Superoxide anion, hydrogen peroxide, the highly active hydroxyl radical, lipid peroxides, and nitrogen oxides are common ROS present in the body (Mailloux, 2015). They are derived from both intrinsic (e.g., electron transport chain, reactions by oxidases) and extrinsic (e.g., UVR, PM2.5) sources. Excessive ROS can directly damage the cellular structure and function, mediate inflammatory responses, and impair genetic components, leading to skin aging (Kammeyer and Luiten, 2015). As a key regulatory target in ROS-induced skin aging, matrix metalloproteinases (MMPs) mediate the degradation of the different components of the ECM (collagen in particular) (Quan et al., 2009). The synthesis of MMPs is stimulated by ROS through the mitogen-activated protein kinase (MAPK) signaling pathway, in which extracellular signal-regulated kinase (ERK), p38, and c-Jun N-terminal kinase (JNK) are common members. The transcription factor, activator protein 1 (AP-1), is then induced and regulates the transcription of MMP-1, MMP-3, MMP-9, and MMP-12 (Shaulian and Karin, 2002). Nuclear factor-κB (NF-κB), another transcription factor, is also activated and mediates the responses to UVR and photoaging by regulating inflammation and MMP expression (Wang et al., 2019a). Thus, MMPs can be modulated by both AP-1 and NF-κB, which makes it a major effector. Another signaling pathway in which MMP is involved in skin aging is the transforming growth factor beta (TGF-β)/Smad pathway, which is impaired upon the downregulation of TβRII expression (partially induced by AP-1) and leads to the reduced production of type I collagen (Quan et al., 2004; Kim et al., 2012).

In the skin, MMPs are primarily secreted by dermal fibroblasts, whereas epidermal keratinocytes are considered the major source of cytokines (Ansel et al., 1990). The expression of MMPs increases in aged skin owing to OS and relative signaling pathways, and tissue inhibitors of metalloproteinases (TIMPs) decrease the level of MMPs (Nagase et al., 2006). Several factors have been reported to fuel ROS-induced aging through upregulated MMPs (Lee et al., 2021a). For example, MMP-9 expression elevation is linked to increased tumor necrosis factor alpha (TNF-α) expression *via* NF-kB, AP-1, hypoxia-inducible factor 1 alpha (HIF-1α), and nuclear factor erythroid 2-related factor 2 (Nrf2) (Holvoet et al., 2003). The accumulation of cysteine-rich protein 61 (CCN1), another cytokine, stimulates the production of MMP1 while downregulating TGF-β type-II receptor, thereby hindering ECM homeostasis during aging (Quan et al., 2011). MMP1 expression can also be enhanced by environmental stressors (e.g., tobacco smoke) *via* the activation of the aryl hydrocarbon receptor (AhR) signaling pathway (Ono et al., 2013).

The over-production of ROS accelerates skin aging. The mitochondria are considered the major source of cellular OS. Endogenous ROS is constantly produced as the byproduct of oxidative phosphorylation in the mitochondrion (Mailloux, 2015). Meanwhile, chronic exposure to UVR also generates ROS, besides increasing nicotinamide adenine dinucleotide phosphate (NADPH) production (Fuller, 2019). During skin aging, ROS also causes damage by inducing mitochondrial dysfunction. Singh *et al.* revealed that the loss of mtDNA homeostasis is responsible for causing skin wrinkles (Singh et al., 2018). This finding was supported by Umbayev *et al.* The authors showed a reduction in fibroblast mitochondrial abundance and mtDNA copy number as well as enhanced mtDNA damage in aged skin (Wei et al., 2016; Umbayev et al., 2020). Besides mtDNA, other evidence indicates associations between the mitochondrial redox imbalance and Nrf2 expression. Jinapath *et al.* confirmed the preventive role of mitochondria-targeted H2S delivery in the mouse skin *in vivo* through Nrf2 activation (Lohakul et al., 2022). The activation of NRF1/NRF2 transcription was also observed in α-l-hexaguluroic acid hexasodium salt-treated HaCaT cells with improved mitochondrial energy metabolism, and subsequently, the increased expression of MMP and silent information regulator 1 (SIRT1) was also observed (Li et al., 2020a). Similar results were noted in HaCaT cells treated with d-tetramannuronic acid tetrasodium salt (Li et al., 2020b). Moreover, the mitochondrial redox imbalance induces JunB proto-oncogene (JunB) expression, causing fibroblast senescence through p16 upregulation and type 1 insulin-like growth factor (IGF-1) downregulation and eventually leading to skin atrophy *via* the disruption of stem cell niches (Maity et al., 2021).

### 2.3 Antioxidants in skin aging

In the past 5 years, several hundred preclinical and clinical studies have shown that the application of antioxidants inhibits skin aging. Herein, we discuss findings from some of the related studies about antioxidants used in treating skin aging to identify potential treatments (Table 1). The anti-aging effect of plant extracts and dietary supplementation has been widely investigated. Additionally, antioxidants extracted from humans and other animals were also shown to be ROS-antagonistic.

**TABLE 1:** Antioxidants in skin aging.

Antioxidants		Anti-ROS activity	Mechanism of anti-ROS activity and others	References
Plant extracts	Artesunate	Anti-ROS	Increased β-catenin expression	Tian et al. (2021)
	Mixture of marigold and rosemary extracts	Anti-ROS, anti-inflammation	Suppression of IL and TNF-α expression, removal of ROS by the restoration of antioxidative enzymes such as SOD, CAT, and GPx	Auh and Madhavan, (2021)
	CSPE nanoformulation	Anti-ROS, anti-inflammation	Downregulation of the mRNA expression of MMP1 *via* the MAPK/C-JNK pathway, increase in collagen and SOD levels, decrease in PGE2, COX2, JNK, MDA and elastin levels	Amer et al. (2021)
	Hydroxytyrosol from olive fruits	Anti-ROS, anti-DNA damage	Inhibition of the formation of 8-dihydroxy-2′-deoxyguanosine (8-OHdG), activation of PCNA, reduction of MMP mRNA expression	Avola et al. (2019)
	Fucoidan isolated from Hizikia fusiforme	Anti-ROS, anti-melanogenesis	Regulation of the ERK–MAPK (extracellular signal regulated kinase-mitogen activated protein kinase) pathway	Wang et al. (2020a)
	Extracts and bioactive compounds derived from seaweeds	Anti-ROS, anti-inflammation, anti-apoptosis	Inhibition of AP-1 and NF-κB expression	Pangestuti et al. (2021)
	Low molecular-weight fucoidan	Anti-ROS, anti-inflammation	Inhibition of the MAPK pathway	Kim et al. (2018)
	BlendE, BlendIP	Anti-ROS, epigenetic modulation	Stimulation of SOD gene expression, modulation of microRNA expression	Namkoong et al. (2018)
	Atractyligenin	Anti-ROS	Inhibition of MAPK pathway, attenuation of c-Fos and c-Jun expression	Xuan et al. (2019)
	BLF	Anti-ROS, anti-inflammation	Regulation of MAPK and autophagy signaling	Gu et al. (2022)
	EGA and DHM	Anti-ROS, anti-inflammation	Possible activation of both TGF-β1 and Wnt signaling pathways	Moon et al. (2018)
	AGE	Anti-ROS, anti-inflammation	Regulation of MAPK/AP-1, NF-κB, and TGFβ/Smad signaling pathways	Jin et al. (2021)
	Salvia haenkei	Anti-ROS	Reduction of IL1α release and ROS generation	Cocetta et al. (2021)
	Hydrangea serrata (Thunb.) Ser. Extract	Anti-ROS	Inhibit AP-1, STAT1, and MAPK signaling pathways	Han et al. (2019)
	Ursolic Acid	Anti-ROS, anti-inflammation, anti-apoptosis	Inhibition of TNF-α-induced MMP activation, suppression of p53 production	Samivel et al. (2020)
	(-)-Loliolide isolated from *Sargassum horneri*	Anti-ROS, anti-inflammation	Suppression of NF-κB and MAPK signaling proteins, downregulation pro-inflammatory cytokines (IL-1β, 6, 8, 33, and TNF-α)	Fernando et al. (2021)
	Tannic acid	Anti-ROS	Inhibition of ROS production and NADPH oxidase activation	Daré et al. (2020)
	Resveratrol	Anti-ROS, anti-inflammation, anti-apoptosis	Inhibition of MAPK and COX-2 signaling pathways, promotion of the Nrf2 signaling pathway, inhibition of caspase activation, upregulation VEGF-B expression	Cui et al. (2022)
	Diphlorethohydroxycarmalol isolated from Ishige okamurae	Anti-ROS	Regulation of NF-κB, AP-1, and MAPK signaling pathways	Wang et al. (2020b)
Dietary supplementation	Ergothioneine	Anti-ROS	Inhibition of the AP-1 pathway and activation of the ARE/Nrf2 pathways	Hseu et al. (2020)
	Walnut protein hydrolysates	Anti-ROS	Modulation of the MAPK/AP-1/MMP-1 and TGF-β/Smad signaling pathways	Xu et al. (2022)
	Caffeine	Anti-ROS, autophagy	Activation of A2AR/SIRT3/AMPK-mediated autophagy	Li et al. (2018)
	Sulforaphane	Anti-ROS	Activation of the Keap1-Nrf2 pathway, macroautophagy/autophagy, and detoxification pathways	Li et al. (2021); Petkovic et al. (2021)
	Alchemilla mollis	Anti-ROS	Regulation of transcription factor NFATc1 and Nrf2/ARE pathways	Hwang et al. (2018)
	Green tea catechin	Anti-ROS	Regulation of NF-κB, AP-1, and MAPK signaling pathways	Wang et al. (2019b)
Endogenous extracts	Melatonin	Anti-ROS, anti-inflammation	Inhibition of hedgehog signaling and inflammatory proteins such as NF-κB/COX-2/ERK/MMP1	Park et al. (2018)
	Growth factors-based platelet lysate	Anti-ROS	Inhibition of the NF-κB signaling pathway	Li et al. (2022)
Animal extracts	Fermented Fish Oil	Anti-ROS	Blockade of the MAPK/AP-1 pathway	Hyun et al. (2019)
Others	Hydrolyzed CTP	Anti-ROS	Anti-ROS, inhibition of glycation	Lee et al. (2022)
	LAB	Anti-ROS	Stabilization of mitochondrial function	Chen et al. (2022)
	30% ethanol extract of EEB	Anti-ROS	Regulation of MAPK/AP-1 and Smad pathways	Choi et al. (2021)

First, antioxidants alleviate skin aging by attenuating OS activity and protecting mitochondrial function. For example, citrus sinensis peel extract (CSPE) nanoformulation (Amer et al., 2021), extracts and bioactive compounds derived from seaweed (Pangestuti et al., 2021), and atractyligenin (Xuan et al., 2019) were reported to suppress ROS by activating the MAPK pathway. As such, CSPE nanoformulations (Amer et al., 2021), BlendE, BlendIP (Namkoong et al., 2018), and tannic acid (Daré et al., 2020) are associated with either an increase in antioxidative enzyme expression or a decrease in ROS generation. Second, the regulation of inflammatory responses is beneficial to anti-aging therapy, such as that with a mixture of marigold and rosemary extracts, CSPE nanoformulation (Amer et al., 2021), bamboo leaf flavonoids (BLF) (Gu et al., 2022), and ursolic acid (Samivel et al., 2020). The level of cytokines is reduced, and other signaling pathways, such as TGF-β and hedgehog signaling pathways, are involved. Third, antioxidant systems exert protective effects on DNA (hydroxytyrosol from olive fruit (Avola et al., 2019)), inhibit apoptosis (ursolic acid (Samivel et al., 2020), resveratrol (Cui et al., 2022)), and promote autophagy (caffeine (Li et al., 2018)).

## 3 Anti-aging effect of stem cells

Stem cells are generally considered powerful candidates in regenerative medicine for combatting skin aging. The disrupted homeostasis and subsequent regulation of ROS activity are considered to be associated with skin aging. Several factors affecting the number, status, and differentiation of stem cells participate in skin aging. Laminins are considered potential anti-aging targets because of their regulatory effects in stem cells. For instance, laminin-332 was confirmed to be responsible for the proper differentiation of interfollicular epidermal stem cells, and the loss of laminin-511 expression results in the reduction of epidermal stem/progenitor cell generation (Yamada et al., 2018; Iriyama et al., 2020). Collagen XVII (COL17A1) showed differential expression in epidermal stem cells in response to genomic or oxidative stress, driving stem cell competition through symmetric cell division. Clones expressing low levels of COL17A1 were eliminated, which promoted skin aging *via* the depletion of adjacent melanocytes and fibroblasts. Meanwhile, the forced maintenance of COL17A1 showed anti-aging potential (Liu et al., 2019). Besides, the crucial role of nuclear receptor interacting protein 1 (Nrip1) in aging was proposed by Hu *et al.* Skin aging was indicated to be delayed with the reduced expression of senescence-associated (p21 and p53), inflammation-associated (p65, IL6, and IL-1α), and growth factor-associated (mTOR, Igf1) genes under Nrip1 knockout in ADMSCs (Hu et al., 2021). The disruption of circadian clock activity through BMAL1 depletion is linked to the increased differentiation of interfollicular epidermal stem cells (IFESCs) in arrhythmic, prematurely aging mice (Welz et al., 2019).

The anti-aging effect of stem cells (including its derivatives) is closely related to their anti-ROS potential, with MAPK and TGF-β signaling pathways (which regulate MMP expression and ECM synthesis) as potential junctions. For example, ADSC-CM was found to prevent photoaging and down-regulate ROS activity through the modulation of the MAPKs/AP-1/NF-κB signaling pathway, with the suppressed expression of MMP-1 and IL-6 and elevated level of antioxidant phase II gene heme oxygenase-1 (HO-1) (Li et al., 2019). Meanwhile, TGF-β and Smad expression was recovered after perturbation by UVB (Li et al., 2019). Additionally, the activation of the NF-κB pathway was also observed in another study conducted by Hwang *et al.* MMP expression and ROS production were ameliorated in the treatment group (with neural stem cell-conditioned medium (NSC-CM) and its secreted factors TIMP-1 and TIMP-2) (Hwang et al., 2019). Moreover, the protective effect of NSC-CM was demonstrated by the activation of the DNA repair enzyme Rad50 and subsequent inhibition of γ-H2AX, a DNA damage marker (Hwang et al., 2019). These results indicate the anti-aging effect of anti-ROS signaling pathways and their promising application of stem cell-conditioned medium.

Besides, other processes, such as the regulation of superoxide dismutase (SOD) expression and fibroblast senescence phenotype by various types of stem cells and their derivatives, are involved. A SOD-dependent mechanism and reversed aging phenotype was suggested by Adam *et al.*, who observed the inhibition of advanced glycation end product and malondialdehyde (MDA, a major product generated by oxygen free radicals) generation and increased SOD expression in their animal model (Zhang et al., 2014; Surowiecka and Strużyna, 2022). Similarly, the injection of ADSC-derived exosomes (ADSC-EVs) observably decreased skin wrinkling, promoted type I collagen synthesis, and suppressed MMP-3 expression (Xu et al., 2020). Evidence from *in vivo* and *in vitro* experiments revealed that ROS production was counteracted with the increased expression of the antioxidant enzymes SOD-1 and CAT (Xu et al., 2020). In another experiment led by Deng *et al.*, hucMSC-derived extracellular vesicles (hucMSC-EVs) suppressed photoaging by inhibiting ROS generation, promoting fibroblast proliferation, and preventing cell cycle arrest, presented with the upregulation of glutathione peroxidase one and Col-1 and downregulation of MMP-1 (Deng et al., 2020). In contrast, the combined use of ADSCs and fractional CO_2_ laser was shown to ameliorate skin aging with a decrease in the MDA content through a SOD-independent pathway (Xu et al., 2014). Xu *et al.* also observed the improvement of cell cycle arrest and increased expression of Wnt3a and β-catenin, which is positively correlated with TGF-β2 and COLI expression (Xu et al., 2014).

Moreover, an adipose tissue extracellular fraction isolated by Barbara *et al.* improved skin aging by reducing OS-induced damage and preventing fibroblast senescence (Bellei et al., 2018). In this process, a slight induction of SESN1 (a p53-responsive protein encoding antioxidant modulators of peroxiredoxins) was observed, indicating the attenuation of ROS (Kopnin et al., 2007; Bellei et al., 2018). Real-time RT-PCR and ELISA analyses conducted by Choi *et al.* revealed the significant suppression of MMP-1, -2, -3, and -9 as well as the enhanced level of collagen and elastin production under the treatment of human adipose-derived stem cell extracellular vesicles (Choi et al., 2019). This was supported by the PCR results reported by Liang *et al.*, which confirmed the increased mRNA expression of type I collagen and decreased expression of type III collagen, MMP-1, and MMP-3 under ADSC-derived exosome treatment (Liang et al., 2020). Simultaneously, TIMP-1 and TGF-β1 were upregulated, which aided the recovery of photo-damaged dermal fibroblasts (Choi et al., 2019).

## 4 Stem cell-based anti-aging treatments

### 4.1 Summary of findings from current clinical trials

Stem cells have shown great potential in skin rejuvenation. Associated treatments include stem cell transplantation and the use of derivatives such as conditioned medium and extracellular vesicles. As a common type of stem cell, mesenchymal stem cells (MSCs) are an essential source for skin rejuvenation and can be categorized into several subpopulations, such as amniotic membrane stem cells (AMSCs), ADSCs, human umbilical cord MSCs (hucMSCs), bone marrow stem cells, and human induced pluripotent stem cells (iPSCs) (Charles-de-Sá et al., 2020). Several clinical trials have been conducted or are underway to address the safety, feasibility, and efficacy of stem cell-based therapeutics to improve skin aging in humans (Table 2). Although some study reports have not yet been published, various clinical trials have evidenced the safety and efficacy of MSCs in this regard.

**TABLE 2:** The clinical trials rendering stem cell-based skin rejuvenation.

Study type	Interventions/treatments	Results	Side effects and limitations	References
An analytic experimental research	AMSC-CM	Significantly better improvement with the AMSC-CM than with normal saline (NS) (*p* < 0.05)	Minor side effects, including erythema for 2 days and urticaria for 3 days; absence of long-term follow-up	Prakoeswa et al. (2019)
A double-blind, split-face, randomized, control study	Protein extracts from medium of ADSCs *via* microneedles	Significantly improved skin roughness, decreased melanin index, increased skin brightness and elasticity, and reduced wrinkles	No adverse reaction observed in 12 weeks	Wang et al. (2018)
A non-randomized, non-blinded study	SVF-enriched fat or expanded ADSCs or fat plus PRP	No significant advantages with the addition of PRP, increased inflammatory infiltration and vascular reactivity	Lack of precise quantification of the changes induced by the treatments, the use of a unique volume ratio (fat/PRP = 1:1), non-randomized study method	Rigotti et al. (2016)
A split-face comparative study	AF-MSC-CM combined with skin needling	Increased epidermal thickness on both sides, greater percentage of improvement, and remodeling of dermal structures on the combined side	Low number of cases, lack of long-term follow-up	El-Domyati et al. (2020)
A randomized controlled trial	*Orobanche rapum* extract	Stimulated skin renewal through protection of skin stem cells and maintenance of skin microbiota balance	Low number of samples	Meunier et al. (2019)
An analytic experimental research	Autologous ADSCs	*De novo* formation of elaunin and oxytalan fibers in the upper papillary dermis, concomitant with degradation of elastotic abnormal elastin deposits in the deeper dermis	No adverse effects observed; limited patient series	Charles-de-Sá et al. (2020)
A double-blind, randomized, vehicle-controlled study	Post-laser treatment with ADSC-CMs in combination with niacinamide	Better skin rejuvenation with decreased levels of pro-inflammatory cytokines and MMP-1 and MMP-2 expression	The need for further research on each component of the product, a relatively short study duration	Lee et al. (2021b)
A prospective, randomized, double-blind, split-face, placebo-controlled study	Red deer mesenchymal stem cell extract	Significant improvement on both sides	The small number of samples	Alhaddad et al. (2019)
A phase one, open-label, single arm study	SVF (containing ADSCs)-enriched fat grafts	Data publicly unavailable at present	Not specified	NCT01828723
A phase 2, randomized, double-blind study	NutraStem	Decreased percentage of CD133+ cells, slightly decreased percentage of CD34^+^ cells, considerably reduced blood level of C-reactive protein	Not specified	NCT01847027

AMSCs are easily obtainable and exhibit low immunogenicity. Also, they secrete several growth factors and cytokines, which can improve collagen synthesis and the proliferation and migration of fibroblasts and keratinocytes (Harrell et al., 2019). Recently, a study on 48 women aged between 41 and 60 years revealed that AMSC-conditioned medium (AMSC-CM) yields significantly lower scores in pore, wrinkle, spot polarized, and spot UV compared to the control group (Prakoeswa et al., 2019). Microneedling was used to enhance the penetration of AMSC-CM. Likewise, the administration of AF-MSC-CM combined with skin needling considerably improved the skin texture, increased collagen and elastic fiber production, and supported the management of facial aging (El-Domyati et al., 2020). According to single-cell profiles, compared with bone marrow stem cells, ADSCs are less heterogenous and less dependent on mitochondrial respiration for energy production, indicating better stemness maintenance and resistance against apoptosis (Zhou et al., 2019).

Evaluation of the potential therapeutic merits of protein extracts (such as IL-6, IL-8, and TGF-β) from ADSCs in 30 Chinese female volunteers showed that, compared with the control group, the intervention group exhibited a considerable improvement in skin roughness, a decreased melanin index, increased skin brightness and elasticity, and reduced wrinkle formation (Wang et al., 2018). Also, an analytical clinical trial of 20 healthy participants (16 women and four men) confirmed the safety and efficacy of autologous ADSC injection in the 3-to-4-month follow-up, as shown by the complete regeneration of solar elastosis (Charles-de-Sá et al., 2020). Further, the remodeling of elaunin and oxytalan fibers in the upper papillary dermis, concomitant with the degradation of elastotic abnormal elastin deposits in the deeper dermis, was observed in the harvested skin samples. Interestingly, an increased level of cathepsin K and MMP-12 and expanded M2 macrophage infiltration were also observed in the post-treatment skin tissues, indicating elastinolysis and the potential anti-inflammatory effects (Charles-de-Sá et al., 2020). Additionally, the application of ADSC-CM (which contains multiple anti-inflammatory cytokines, growth factors, and ECM-regulating molecules) in combination with niacinamide resulted in a statistically more drastic improvement of wrinkles and skin pigmentation in patients post-laser therapy (Lee et al., 2021b).

Moreover, the results of the *in vitro* analysis suggested the upregulation of type I collagen and decreased pro-inflammatory cytokine, MMP-1, and MMP-2 expression in a dose-dependent manner (Lee et al., 2021b). To compare the regenerative effects of platelet-rich plasma (PRP) injection and ADSCs, 13 patients were injected with stromal vascular fraction (SVF)-enriched fat, expanded adipose-derived stem cells, or fat plus PRP (Rigotti et al., 2016). In addition to findings from their previous study, Gino *et al.* showed the revearsal of aging in collagen and elastin morphology (Charles-de-Sá et al., 2015; Cohen, 2016). However, the addition of PRP led to no significant advantages but increased inflammatory infiltration and vascular reactivity (Rigotti et al., 2016).

Red deer mesenchymal stem cell extract, another source of stem cells, also showed the ability to rejuvenate aging facial skin with acceptable safety and feasibility (Alhaddad et al., 2019). Besides, some forms of skin rejuvenation occur along with an increase in the level of stem cells. For example, *O. rapum* extract was demonstrated to reactivate skin renewal *via* the protection of skin stem cells and maintenance of skin microbiota balance (Meunier et al., 2019). While the level of peripheral CD34^+^ and CD133+ stem cells failed to increase under the treatment of NutraStem^®^ in combination with an exercise stimulus, a relative restorative potential and anti-oxidative effect was implied. In addition, no severe side effects were observed among the treated groups, which indicated the safety and feasibility of stem cell-based therapy. The common limitations of such studies include the small sample size and lack of long-term follow-up.

### 4.2 Promising applications of nano-materials: Nanofat and stem cell-derived extracellular vesicles

As mentioned above, skin aging is an extremely complex process that is primarily induced by OS and other intrinsic and environmental factors. In this process, stem cell homeostasis exhibits significant anti-ROS potential in delaying the aging process. The primary goal of skin rejuvenation is to help prevent serious diseases related to skin aging (such as skin cancers) as well as to meet the psychological and aesthetic needs of patients.

Classic options include plastic surgeries, infrared therapy, topical medication (i.e., lotions, injections, and fillers), and dietary supplements (Tsai and Hamblin, 2017; Wang et al., 2020c; Geahchan et al., 2022). Although some of them have been confirmed to be effective, many patients undergoing these treatments complain of limited improvement and lengthy therapeutic cycles. Thus, the development of novel technologies is required. Nanosized ingredients offer enormous advantages, such as better permeation across skin layers, to mediate the intended anti-aging effects (Bhatia et al., 2022). Here, we will discuss the development of combined nanotechnology with stem cells over the last decade, with an aim of providing a useful framework of stem cell-related nano-products.

Generally, nanofat and stem cell (especially ADSC)-derived extracellular vesicles (EVs) (primarily exosomes) are the two core components used. With respect to nanofat, single grafting as well as integrated use with other techniques such as microneedling were invented. When the dimensions are 400–600 μm or less, nanofats include micro-fragmented adipose tissue-containing matrix, stromal vascular cells, and free fatty acids. This can be easily obtained through emulsification and injected through a 27-gauge needle or smaller needle, which is considered an *in vivo* tissue-engineering treatment (Cohen et al., 2017). As adipocytes cannot survive the isolation process, enhanced stem cell activity is probably a major effect.

For example, the enrichment of ADSCs in nanofat samples was observed by Patrick *et al.* The subsequent 67 cases treated with nanofat grafting reported improved skin quality with reduced rhytides and pigmentation (Tonnard et al., 2013). Similarly, the combined use of microfat and nanofat grafting yielded satisfactory results in lower eyelid dark circles, indicating the stem cell-like activity of nanofat as potential therapy for skin rejuvenation (Oh et al., 2014). The nanofat group exhibited significantly improved facial soft tissue depression and skin texture as well as an overall satisfaction rate above 90% (Wei et al., 2017). Nanofat-derived stem cells, which show enhanced proliferation and adipogenic differentiation induced by platelet-rich fibrin, functioned similarly to MSCs and shared many of the biological characteristics, such as high levels of CD29, CD44, CD49days, CD54, CD90, and CD105 expression and low levels of CD34, CD45, and CD106 expression (Wei et al., 2017). While microneedling alone requires repeated treatment, its incorporation with nanofat yielded a more lasting anti-aging effect (Verpaele et al., 2019).

The second strategy used for stem cell-derived EV treatment refers to delivery and regulation through nanosized vesicles. Common sources include human umbilical cord mesenchymal stem cells, ADSCs, and iPSCs. EVs are lipid-bilayer carriers (such as exosomes, microvesicles, and apoptotic bodies) containing proteins, lipids, RNAs, and DNAs, responsible for intercellular communication (Théry et al., 2018). As a major subtype of EVs, exosomes are nanoparticles with a diameter of 40–150 nm, known for their ability to deliver not only numerous proteins (e.g., enzymes, cytokines, and transcription factors) but also nucleic acids (especially miRNAs) (Cha et al., 2020). Therefore, stem cell-derived exosomes have shown great potential in skin aging treatment by enhancing fibroblast proliferation and bioactivity, reducing ROS and inflammation, upregulating collagen expression, and downregulating MMP expression. For example, the treatment of exosomes derived from hucMSCs (hucMSC-ex) promoted H_2_O_2_ detoxification, repressed DNA damage, and inhibited apoptosis, leading to the attenuation of skin redness, scaling, and inflammatory cell infiltration. Additionally, the cytoprotective effects of hucMSC-ex-derived 14-3-3ζ protein might be associated with the modulation of the antioxidant SIRT1-dependent pathway (Wu et al., 2021). Besides, the anti-aging effect of hucMSC-ex can be strengthened through combination with the marine sponge *Haliclona* sp. *Spicules* (SHSs) (Zhang et al., 2020), which resulted in the promotion of HDF proliferation, reduction in the proportion of senescent cells, and rebuilding of the dermal ECM (Zhang et al., 2020). Similarities with remodeled ECM are visible in corneal stromal cells treated with ADSC-derived exosomes (Shen et al., 2018). Besides, Oh *et al.* reported the suppression of SA-β-Gal and MMP-1/3 and restored type I collagen expression in senescent HDFs treated with exosomes derived from human iPSCs (iPSCs-Exo) (Oh et al., 2018).

Recently, a novel bioinspired approach, cell-engineered nanovesicles (CENVs), with diameters less than 150 nm, was proposed for treating issues like the inhibited production of exosomes while maintaining similar characteristics. In addition to the above changes, iPSC-CENV also inhibited the elevation of p53 and p21 expression, indicating the potential modulation of cell cycle arrest, apoptosis, and cellular senescence (Lee et al., 2020).

## 5 Discussion

As the human life span extends, the need of people for anti-aging treatment increases. As the direct manifestation of aging, skin aging is a major concern for health-related and aesthetic reasons. It is caused by a joint attack by genetic and environmental factors and can lead to consequences such as elevated risks of injuries, infection, impaired wound healing, and cancer. Thus, it is important to identify methods to delay skin aging.

Chronological and extrinsic skin aging exhibit distinct differences in clinical indicators but involve similar regulatory pathways. In the past few years, numerous studies have been conducted in the field of skin rejuvenation, and some have achieved promising results. OS is considered to initiate skin aging. The overproduction of ROS can induce mitochondrial dysfunction, inflammation, DNA damage and ECM alteration *via* the activation of MAPK and TGF-β signaling pathways, leading to the upregulation of MMP expression and suppression of collagen synthesis. Antioxidants, including dietary supplements and extracts from plants and animals, ameliorate ROS production, and their therapeutic effects have been confirmed in animal models. Owing to their convenience, easy accessibility and portability, and relatively lower prices, antioxidants are considered as potential treatments. This also led us to determine whether the use of antioxidants from an early age can reduce or prevent further harm. The ingredients need to be used for extended periods to ensure visible improvement and may require lifetime intake/application. Meanwhile, their safety and anti-aging effects need further confirmation.

Stem cell-based treatment has shown great potential in skin rejuvenation and regenerative medicine, as evidenced by findings from several clinical trials. Stem cells such as ADSCs protect cells from oxidative damage by secreting growth factors (such as HGF and VEGF), cytokines such as IL-6 (through the promotion of STAT3 and Nrf2 expression), and antioxidant enzymes (eg. GPx, SOD, and catalase). Protective factors such as laminins, COL17A1, Nrip1, and BAML1 participate in skin aging by promoting stem cell homeostasis. Therefore, it is likely that these factors can serve as anti-aging targets. In addition, the recent progressive application of stem cell-based nanotechnologies has provided insights on this issue. Physical and histological therapeutic effects were observed in nanofat grafting and exosomes derived from stem cells, some of these therapeutic effects were observed in human. Skin rejuvenation yields more significant effects in a shorter time span. However, many of the procedures are invasive and can only be performed at certain qualified facilities. Besides, challenges involving healthcare regulatory issues, poor survival of administered cells, and the risk of biological contamination are present. Furthermore, a cell-free system, such as the sustained release of adipose collagen fragments, has been suggested to overcome the disadvantages of stem cell treatment. Based on the minor complications reported, the safety and feasibility of stem cell-based therapy need to be confirmed in a larger population.
